# Personalized Digital Health Communications to Increase COVID-19 Vaccination in Underserved Populations: A Double Diamond Approach to Behavioral Design

**DOI:** 10.3389/fdgth.2022.831093

**Published:** 2022-04-15

**Authors:** Kelsey Lynett Ford, Ashley B. West, Amy Bucher, Chandra Y. Osborn

**Affiliations:** Lirio LLC, Franklin, TN, United States

**Keywords:** health equity (MeSH), digital health (eHealth), personalization, behavioral science, health communication (MESH), behavioral design

## Abstract

The COVID-19 pandemic exacerbated pre-existing health disparities. People of historically underserved communities, including racial and ethnic minority groups and people with lower incomes and educational attainments, experienced disproportionate premature mortality, access to healthcare, and vaccination acceptance and adoption. At the same time, the pandemic increased reliance on digital devices, offering a unique opportunity to leverage digital communication channels to address health inequities, particularly related to COVID-19 vaccination. We offer a real-world, systematic approach to designing personalized behavior change email and text messaging interventions that address individual barriers with evidence-based behavioral science inclusive of underserved populations. Integrating design processes such as the Double Diamond model with evidence-based behavioral science intervention development offers a unique opportunity to create equitable interventions. Further, leveraging behavior change artificial intelligence (AI) capabilities allows for both personalizing and automating that personalization to address barriers to COVID-19 vaccination at scale. The result is an intervention whose broad component library meets the needs of a diverse population and whose technology can deliver the right components for each individual.

## Introduction

People of all ages and races/ethnicities across socioeconomic strata were accessing and using mobile devices (e.g., cell phones, tablets, laptops) and their applications well before the COVID-19 pandemic (1, 2). The public health recommendation to social distance during the pandemic increased dependence on mobile devices for work, household maintenance, and social connections (3). People relied more on digital channels (e.g., email, text messaging) and mobile applications for maintaining relationships (e.g., Facebook), working (e.g., Zoom, Teams), shopping (e.g., Amazon), managing their health, and accessing health care (i.e., via telehealth) (4).

Also, in the United States, the pandemic brought well-established health disparities to the forefront. Vast evidence demonstrates racial/ethnic minority groups and/or those with a lower income, less education, or inadequate health insurance have worse health, limited health care, and higher rates of premature mortality relative to Whites and those of higher socioeconomic status (SES) (5). COVID-19 exacerbated these health inequities as racial/ethnic minorities and members of lower SES groups were significantly more likely to experience COVID-19 infections, hospitalizations, and COVID-19-related premature mortality compared to their White and higher SES counterparts (5).

Although COVID-19 vaccinations are effective at preventing COVID-19 hospitalization and death, fewer Blacks have been vaccinated against COVID-19 compared to White Americans (6, 7). In March 2021, three months into vaccine distribution, an absolute 6% more Whites than Blacks had been vaccinated in 43 US states. In November 2021, this disparity held with an absolute 7% more Whites (56%) than Blacks (49%) vaccinated against COVID-19 (8).

COVID-19 vaccination promotion efforts produce varying results, with few effectively closing disparities in vaccination rates (9). Personalized approaches such as 1:1 provider and patient conversations are successful, yet resource intensive (10). Digitally-delivered (via email and text messaging) interventions have been shown to effectively reach and engage lower those of lower socio-economic status (11–14). Additionally, interventions overcoming each person's barriers to doing a health behavior are effective across all populations (e.g., racial/ethnic minorities, individuals with low SES, and/or those with low health literacy) (15, 16). Promise remains in identifying and intervening on barriers of COVID-19 vaccination among diverse populations (17). To truly close gaps in health disparities, the challenge remains to provide initial and ongoing digital personalization in an equitable, automated, scalable way (18, 19).

To create scalable, equitable interventions for behavior change challenges like encouraging vaccination, we propose intentional behavioral design using the Double Diamond model of innovation to inclusively identify determinants (i.e., barriers and facilitators) for a recommended behavior, creating an evidence-based digital intervention to address those determinants, and harnessing behavior change artificial intelligence (AI) capabilities to automate and personalize the intervention to each individual within the population. Here, we share an example of a behavioral design process and behavior change AI application to address barriers to COVID-19 vaccination for the purpose of promoting the vaccine at scale.

## Intentional and Inclusive Behavioral Design

**Figure 1:**
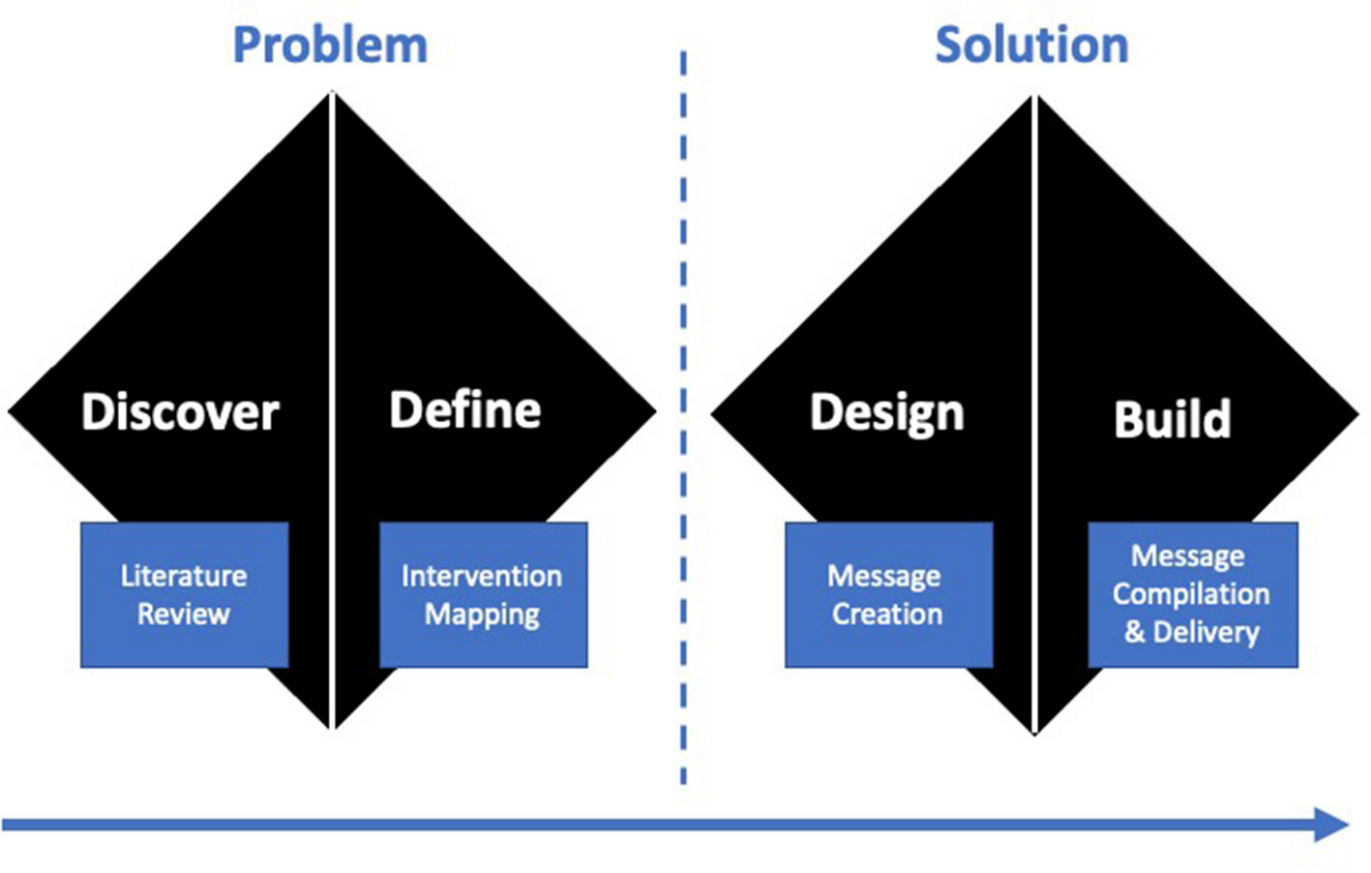
Consistent with the UK Design Council's Double Diamond model (i.e., discover-define-develop-deliver), behavioral science can be interwoven into the model's best practice methods that alternate between divergent stages, where the focus is on gathering information and thinking broadly, and convergent stages, where the focus is on prioritization and refinement.

Behavioral science can be combined with well-established design processes such as the Double Diamond model (20) for more intentional intervention development, resulting in interventions that improve outcomes for all, particularly underserved populations (21). The Double Diamond model blends user-centered design principles into scientific intervention development by alternating exploration and solutioning stages across the development process (Figure 1).

In our vaccination promotion efforts, behavioral designers adhered to the Double Diamond model during intervention development to equitably promote COVID-19 vaccinations across diverse populations. Our intervention is an AI-driven behavior change platform that uses AI to select, assemble, and send message components from a behavioral science-based library to drive target behaviors. The target behaviors were scheduling the COVID-19 vaccination(s) and completing scheduled appointments. Based on recipients' actions, including interacting with the message, scheduling, and attending vaccination appointments, the intervention platform can further personalize subsequent messages to increase the frequency of target behaviors.

Designers performed a scientific literature review to identify the behavioral determinants (i.e., barriers and facilitators) for the above target behaviors. Then, as a best practice in behavioral science, designers employed an Intervention Mapping (21) process linking determinants to evidence-based behavior change techniques (BCTs) (22). Designers created a library of behavioral science message components (i.e., subject lines, body copy) and visuals suitable for both email and text messaging channels. Lastly, messages were assembled from components and delivered using a behavior change AI platform designed to personalize messages based on individual behaviors and characteristics.

Recipients were diverse in terms of race, ethnicity, income and education levels, and other demographics. The phases of the Double Diamond model (i.e., Discover, Define, Develop, Deliver) allowed for intentional and inclusive behavioral design, ensuring that library components included evidence-based behavior change strategies for the specific behavioral determinants prevalent across sub-populations, and that the AI technology was able to select the appropriate components for a given individual. To align with this objective, we outline the activities for each phase of the Double Diamond model.

### Discover

The objective of the Discover phase is to deeply understand the behavior change problem. Behavioral designers searched scientific databases (e.g., PubMed, EMBASE) and relevant, credible gray literature (e.g., Pew Internet Research data, other national polls) to cast the widest net of documented historical and current determinants for vaccination adoption. Designers included both COVID-19 vaccination research and the evidence base for other communicable viruses (e.g., flu, HPV). Initiating the behavioral design process with an expansive list of determinants ensures intervention design remains inclusive for diverse populations.

In instances when the behavioral science literature is too new, as with COVID-19, to be comprehensive, partnering and learning from stakeholders who understand the behavior change problem and can provide insight into the determinants is recommended. We consulted with a variety of stakeholders specializing in population health, community health, and healthcare more broadly who provided insight into the determinants of both patient vaccination adoption in general and COVID-19 vaccination adoption, specifically.

### Define

The objective of the Define phase is to analyze and synthesize learnings from the Discover phase into themes, priorities, and design requirements. Approximately 80 publication sources revealed at least 45 determinants related to vaccination adoption (23, 24). Of these determinants, roughly 10 barriers were particularly prevalent among historically underserved populations, including people with lower SES, people residing in rural areas, and racial/ethnic minorities (Table 1).

**Table 1:** Unique behavioral determinants exist for vaccination adoption among historically underserved populations (*n* = 10).

Difficulty understanding health information
Unknowns about vaccine distribution, delivery and implementation
Believing in conspiracies
Negative emotions (e.g., confusion, nervousness, apathy, anger, fear)
Concern about vaccine access
Concern about vaccine cost
Concern about vaccine safety (e.g., side effects, distrust of medical institutions)
Concern about vaccine efficacy
Underestimating risk associated with opting out of vaccination
Dislike being told what to do by the government/other authorities

*Our literature review captured 10 key barriers among people with a lower SES, living in rural areas, and racial/ethnic minorities, including concern about vaccine access, concern about vaccine safety and efficacy, and underestimating risk associated with opting out of the vaccine*.

In the Define phase, designers categorized and prioritized vaccination barriers for all populations. Designers flagged barriers most relevant to underserved populations as critical for inclusion in intervention design. Designers defined intervention features to specifically target these and other prevalent barriers and specified relevant demographic information to be captured to better personalize the messages.

As part of the Define phase, we identified mechanisms of action and then BCTs to best support people overcoming barriers to vaccination, paying special attention to nuances required to support the underserved. For example, racial and ethnic minority communities may have concerns about historical and unethical medical research. Leveraging credible sources is an effective behavioral strategy for changing an individual's attitudes about a health behavior. However, when using this BCT to support racial and ethnic minorities in this COVID-19 context, the use of a trusted community member as the credible source may be better received than assuming the credible source is a healthcare provider.

### Develop

In the Develop phase, designers assembled requirements and created intervention content and features. The intervention content was a deliberately broad message component library written to be inclusive of the needs of a diverse population of recipients, including members of underserved communities.

Behavioral designers utilized the behavior change taxonomy and COM-B model to map determinants to mechanisms of action and then selected a single, effective behavior change technique (BCT) to address each one (25, 26). BCTs were selected based on both the strength of evidence for addressing the determinant and the ability to translate the BCT into a digital communication. Further, we carefully considered whether a particular determinant deserved to be treated separately (i.e., received its own BCT and message components), rather than consolidated with similar determinants, due to its importance to and impact on specific groups.

A creative team operationalized each BCT into a text and visual component for email and text channels that was written and displayed at a 5–8th grade reading level (27–29) to accommodate recipients with limited literacy.

### Deliver

The final phase of design is Delivery, where advanced consumer technologies can use what is known about an individual to personalize intervention delivery. This allows for the selection of BCTs appropriate for an individual's needs from the broader library designed to accommodate a diverse population, as well as feedback-driven course correction based on recipient behavior. In this way, technology can address the issue that while underserved communities may experience some barriers at a greater intensity or frequency than the population at large, an individual within that community may not experience those specific barriers. In general, personalization can enhance intervention efficacy: personalized interventions or messages are more individually-relevant and, therefore, more efficacious for driving engagement, comprehension, and action (30). For our intervention, the use of an AI platform was essential to achieve this level of personalization.

#### Personalize and Scale With Behavior Change AI

Our team leveraged a proprietary AI reinforcement learning platform to select, assemble, and deliver the right message components for individuals based on their characteristics and behaviors. The reinforcement learning was designed to reward the AI agent for messages that yielded behavioral outcomes (i.e., opening messages, scheduling vaccination appointments, or completing vaccination appointments), so that it becomes increasingly accurate in selecting the BCTs that work for a particular individual as they interact with the intervention.

Messages were delivered via email or text message, based on recipient's available contact information and communication preference. Accommodating communication preferences is critical to an intervention's success (31). Moreover, communication channels that do not rely on broadband internet access (e.g., text message) are recommended to reach underserved populations such as people living in rural areas and racial and ethnic minorities (32). Recipients received messages via their preferred digital communication channel (i.e., email or text message) at a cadence of one personalized message per week for five weeks, followed by a break in communications to avoid notification fatigue (33). Messages ceased if the patient scheduled a COVID-19 vaccination appointment or unsubscribed from messaging.

#### Iterative Improvements

Real-world, just-in-time intervention improvements is a notable, persistent behavioral science challenge (34), but was necessary for this intervention to succeed as the pandemic changed over time. Designers updated the intervention at regular intervals to remain contextually relevant. These updates specifically re-evaluated known determinants for continued relevance and reviewed content for continued accuracy. For example, later versions of the messages promoted autonomy, to be particularly mindful of individuals who might be increasingly more unwilling to be vaccinated. Another example accompanied the rollback of mask requirements in many areas; we updated message visuals to include a variety of masked and unmasked figures, and updated language to de-emphasize mask wearing as an expected behavior in public contexts. A review of the behavioral data before and after these intervention updates suggests no disruption to AI agent learning or intervention effectiveness.

## Discussion

Intentional behavioral design that leverages evidence-based behavioral science intervention techniques, the Double Diamond model, and behavior change AI technology can address barriers to COVID-19 vaccination hesitancy, offering a way to inclusively target determinants for underserved populations and deliver behavior change messages at scale. Personalized interventions delivered through digital consumer channels have the potential to meaningfully address vaccination hesitancy at the individual level, while accommodating a broad set of behavioral determinants inclusive of those experienced by underserved populations. This blend of behavioral science and the Double Diamond model extends the best practices offered in other digital health equity recommendations into the specific realm of intervention design (31, 35, 36). We offer three critical design considerations for any behavior change intervention strategy that aims to achieve personalization and digital health equity.

First, it is vital to understand and account for everyone's barriers and facilitators to achieving the recommended health behavior, which may necessitate iterating the intervention as the recipients, behavioral determinants, and context changes over time (i.e., Discover phase). To promote health equity, behavioral design efforts must deliberately consider a broad range of behavioral determinants, contextual constraints and opportunities, and the importance of personalization. The Double Diamond's Discover phase can include conducting a broad and inclusive literature review (i.e., theoretical, historical, and practical) of the problem, its impact on health equity, and determinants of both. In doing this, it is critical to understand the origins of disparities and challenges (e.g., context, resources, barriers, facilitators) specific to social determinants of health (18, 37).

Second, once identified, designers should match the appropriate behavior change techniques to address each of the identified determinants. Each message should operationalize an evidence-based BCT (i.e., Define and Develop phase). While this requires careful translation of BCTs into the intervention medium, it also means aligning design with real-world constraints and needs. This is where partnering with stakeholders can inform the overall and equitable aspects of an intervention's design, and real-world implementation. Meaningful involvement from stakeholders (e.g., health system leadership, leaders from underserved communities) is recommended to ensure appropriate intervention design (31). Input from individuals of underserved communities holds incredible value (31), however remains rare in digital health endeavors.

Third, if feasible, technology such as behavior change AI should be applied to personalize the messages at scale during the Deliver phase. The use of AI and advanced technical capabilities offers an opportunity to achieve population-level reach with individualized content. Behavioral “nudges” distributed to diverse populations has increasing potential to promote health equity (17, 36). The more an intervention can be personalized to its recipients, the more likely it is to succeed and sustain behavior change (38). Additionally, personalization for multiple characteristics (e.g., preferences, channels, context) of an intervention enhances intervention efficacy (32, 39), and more complex personalization may require more technology support.

While AI is a relatively new tool in behavioral interventions, researchers, designers, and interventionists have already found utility in using AI to deliver context-aware digital behavior change interventions (40, 41). The use of automating technologies to leverage high-volume data points such as user inputs, behaviors, geotags, or sensor measurements may accelerate health equity efforts to ensure context is considered in digital behavior change intervention development (40). At the same time, technology that can consume and interpret such complex real-world data will yield a more accurate and complete representation of its users, supporting the design and delivery of more appropriate intervention content.

Real-world considerations are critical pieces in the behavioral design of inclusive interventions, informing iteration and implementation. As real-world contexts evolve, people's barriers and determinants change. It becomes an ethical duty for designers to improve their interventions accordingly with thoughtful design processes. Anticipating iterative improvements after implementation ensures content remains relevant and meaningful to all, and more likely to promote engagement and action with underserved populations.

Although digital health interventions have existed for decades, the marriage of behavioral scientists and traditional design teams is relatively new and can present challenges as professionals with different training and focus areas working together to build digital tools. Integrating intervention development with the Double Diamond model facilitates a new form of collaboration between behavioral scientists, designers, and technologists to create equitable behavior change interventions. We believe this aligned approach will facilitate more productive design of interventions that are both efficacious, engaging, and activating.

Despite the innovative integration of behavioral science to the Double Diamond model, limitations exist when leveraging behavioral design and AI in a real-world context. This intervention prioritized two widely used channels that are particularly accessible to members of underserved populations (i.e., email and text), but other intervention touchpoints such as chatbots or in-app notifications may be just as or more effective for some people, behaviors, and contexts (42). Moreover, any real-world implementation involves factors outside of designers' control. In this case, the lack of an accessible, unified vaccination database in the United States means interventions almost certainly undercount recipients who successfully completed their COVID-19 vaccination series. This undercount limits research insights into effective behavior change techniques for all and specifically for underserved populations. As we can augment behavioral data with additional sources, we can close this gap.

In conclusion, the COVID-19 pandemic necessitated a swift and inclusive population-based public health response. The pandemic and associated vaccination rollout emphasized health disparities, unique individual attitudes and beliefs, and the need for scalable interventions. Digital interventions, when informed by evidence-based behavioral science and design processes, offer an opportunity to engage and reach a diverse audience. The current intervention utilized the Double Diamond model to both create a COVID-19 vaccination intervention informed by a diverse set of resources (e.g., peer-review and gray literature, stakeholder feedback) and intentionally adapt to allow for in-tandem design and development responsive to the evolving pandemic. In addition, behavior change AI technology allowed for the delivery of personalized communications at scale. The combination of evidence-based behavioral design and automated personalization sets the stage for closing disparity gaps in COVID-19 vaccination at scale, and provides a template for how designers can close disparity gaps across a variety of health behaviors.

## Data Availability Statement

The original contributions presented in the study are included in the article/supplementary material, further inquiries can be directed to the corresponding author/s.

## Author Contributions

KF, AW, and AB wrote the first draft of the manuscript. CO and KF revised the manuscript. All authors wrote subsections of the manuscript. All authors contributed to the article and approved the submitted version.

## Funding

This study received funding from Lirio. The funder was not involved in the study design, collection, analysis, interpretation of data, the writing of this article or the decision to submit it for publication.

## Conflict of Interest

KF, AW, AB, and CO were employed by Lirio LLC.

## Publisher's Note

All claims expressed in this article are solely those of the authors and do not necessarily represent those of their affiliated organizations, or those of the publisher, the editors and the reviewers. Any product that may be evaluated in this article, or claim that may be made by its manufacturer, is not guaranteed or endorsed by the publisher.
